# Mechanism-based modeling of the effect of a novel inhibitor of vascular adhesion protein-1 on albuminuria and renal function markers in patients with diabetic kidney disease

**DOI:** 10.1007/s10928-020-09716-x

**Published:** 2020-09-14

**Authors:** Sven Hoefman, Nelleke Snelder, Martijn van Noort, Alberto Garcia-Hernandez, Hartmut Onkels, Tobias E. Larsson, Kirsten R. Bergmann

**Affiliations:** 1 LAP&P Consultants BV, Archimedesweg 31, 2333 CM Leiden, The Netherlands; 2 Astellas Pharma Europe BV, Global Development, Sylviusweg 62, 2333 BE Leiden, The Netherlands

**Keywords:** VAP-1 inhibition, Albuminuria, Mechanism-based modeling

## Abstract

The vascular adhesion protein-1 (VAP-1) inhibitor ASP8232 reduces albuminuria in patients with type 2 diabetes and chronic kidney disease. A mechanism-based model was developed to quantify the effects of ASP8232 on renal markers from a placebo-controlled Phase 2 study in diabetic kidney disease with 12 weeks of ASP8232 treatment. The model incorporated the available pharmacokinetic, pharmacodynamic (plasma VAP-1 concentration and activity), serum and urine creatinine, serum cystatin C, albumin excretion rate, urinary albumin-to-creatinine ratio, and urine volume information in an integrated manner. Drug-independent time-varying changes and different drug effects could be quantified for these markers using the model. Through simulations, this model provided the opportunity to dissect the relationship and longitudinal association between the estimated glomerular filtration rate and albuminuria and to quantify the pharmacological effects of ASP8232. The developed drug-independent model may be useful as a starting point for other compounds affecting the same biomarkers in a similar time scale.

**Electronic supplementary material:**

The online version of this article (10.1007/s10928-020-09716-x) contains supplementary material, which is available to authorized users.

## Introduction

Albuminuria is a surrogate marker of progression of chronic kidney disease and a reduction of albuminuria is associated with improvements in long-term clinical outcomes, such as end-stage renal disease. It is currently under evaluation whether albuminuria-lowering compounds can aid to lower the rate of renal filtration loss in conjunction with antihypertensive therapy [1, 2]. ASP8232 is a small molecule Vascular Adhesion Protein-1 (VAP-1) inhibitor that demonstrated an albuminuria lowering effect in a Phase 2 trial in diabetic kidney disease (DKD) patients (ALBUM study) [3]. Following a 12-week treatment period with 40 mg qd ASP8232, the primary and key secondary endpoints indicated a 20% reduction in 24-h urinary albumin concentrations and a 19.5% reduction in first morning void (FMV) urinary albumin-to-creatinine ratio (UACR) *versus* placebo, respectively. These effects were thought to result from the near-complete inhibition of VAP-1 activity during treatment. Indeed, soluble VAP-1 levels are increased in subjects with diabetes and early stages of chronic kidney disease, and its oxidase activity is expected to play a pathogenic role in DKD patients [4–7].

In the ALBUM study, serum creatinine (sCr), which is a common marker for the estimated glomerular filtration rate (eGFR), increased upon ASP8232 treatment [3]. Interestingly, increases in serum cystatin C (sCysC), another marker for renal filtration, were much less pronounced. CysC is a 13.3 kDa protein that is pre-dominantly filtered by the kidneys and subsequently degraded following uptake by proximal tubular endothelial cells [8–10]. Unlike sCr, sCysC is not actively secreted [10–12]. The observed rise in sCr levels, and thus the decrease in sCr-based eGFR, is consistent with an inhibition of transporters (MATE1, MATE2-K, and OCT2) involved in sCr secretion, as previously reported and supported by preclinical and phase 1 clinical data [3]. The small and reversible reduction in eGFR CysC observed during ASP8232 treatment, indicates that ASP8232 has acute haemodynamic effects [3].

Non-linear mixed effects (NLME) exposure–response (ER) models are used to establish the quantitative link between drug concentration and effects on an individual and population level [13]. These models can be used to explain the effects of drugs on a biological system with varying levels of complexity [14, 15]. Keeping assumptions and limitations in mind, the models can be useful to predict the outcome of untested situations and are thus valuable assets in the clinical development process [16]. In a companion paper [#], a NLME pharmacokinetic-pharmacodynamic (PK-PD) model has been developed for ASP8232. This model was able to describe the absorption and disposition of ASP8232, as well as its interaction with soluble and membrane bound VAP-1 and its inhibition of VAP-1 activity.

In the present study, an ER model was developed to describe the effect of ASP8232 on GFR, albuminuria and sCr in DKD patients. This was achieved by simultaneously incorporating sCysC, sCr, albumin excretion rate (AER), UACR, urine creatinine (uCr) and urine volume data in the model, using dose or individual ASP8232 plasma concentrations not bound to VAP-1 (C_u_) obtained from the ASP8232 PK-PD model as driver of the effects. The main objectives of this analysis were to quantify the relationship and longitudinal association between GFR and albuminuria, investigate early signs of an ASP8232 effect on the chronic GFR slope, uncouple the GFR mediated and direct ASP8232 effects on albuminuria, and uncouple the effects of change in GFR and ASP8232 transporter inhibition on sCr.

## Methods

### Study design

The model was developed based on data from a Phase 2 trial in DKD patients. This study was a double-blind, randomized, parallel-group, placebo-controlled, multi-site study (ClinicalTrials.gov NCT02358096) [3]. The study consisted of a 1-week screening, 5-week pre-treatment, 12-week treatment and 24-week follow-up period. During the treatment period, patients received oral 40 mg ASP8232 or placebo once daily (qd). Further study details are provided in the article by de Zeeuw et al. (2018) [3].

### Sampling and measurements

During the 12-week treatment period, samples at a given visit were collected prior to the dose. sCysC and sCr were measured at the screening and baseline visits and at week 2, 4, 6, 8, 12, 16, 24 and 36. AER, uCr and urine volume were measured based on 24 h samples collected at baseline, week 4 and 12. UACR FMV samples were collected at the screening and baseline visit and at week 2, 4, 6, 8, 12, 16, 24 and 36. At screening, one FMV UACR sample was taken. During pre-treatment, six FMV UACR samples were taken, which were assigned to the baseline visit. For subsequent visits, three FMV samples were collected as follows: 2 days before, 1 day before, and on the morning of the scheduled site visit, except for the week 4 and 12 visits, when the subject collected the FMV sample 3 days, 2 days and 1 day before the site visit. In the ER analysis, actual collection time and measured concentration was used for each sample, without summarizing the triplicated FMV measurements. Therefore, assignment to any visit number did not have any impact.

Urinary albumin, sCr and uCr concentrations were measured as described by de Zeeuw et al. (2018) [3]. sCysC concentrations were determined using a validated particle enhanced immunonephelometric assay on a Behring BNII nephelometer (CC-EQ-330-CHIM) and a commercial kit (N Latex cystatin C, Cat. Nr: OQNM, Dade Behring, Marburg, Germany). The sensitivity of the urinary albumin assay was 3 mg/L (for other variables, all measurements could be quantified). Assay performance was according to the assay manufacturer’s packaging inserts.

eGFR by sCysC (eGFR CysC; mL/min/1.73m^2^) was derived according to Inker et al. (2012) [17], from the sCysC values and each individual’s age and sex. AER (mg/24 h) was calculated by multiplying the urinary albumin concentration with the urine volume sampled over 24 h. UACR (mg/g) was calculated by dividing the urinary albumin by the uCr concentration in FMV samples.

In total, 1164 eGFR sCysC, 1174 sCr, 3552 UACR, 346 AER, 349 uCr and 348 urine volume measurements were available for analysis for 120 DKD patients (60 ASP8232-treated and 60 placebo patients). One AER value and seven UACR values were reported to be below the detection limit and were excluded from the analysis. During model development, exclusion of outliers from further analysis was allowed, defined as conditional weighted residuals > 5 or <  − 5, if this led to a stabilization of the model. Based on the PK-VAP-1 model described in a companion paper [#], the model-predicted unbound ASP8232 concentrations, C_u_, at the observation time points were added to the dataset for ER modeling.

### Main modeling assumptions

- eGFR CysC was assumed to be a good proxy for the true GFR (which is not measured).
- eGFR CysC was assumed not to be affected by changes in albuminuria or sCr.
- An increase in GFR, characterized by an increase in eGFR CysC, was assumed to lead to a decrease in sCr and increase in AER, and vice versa. Similarly, subjects with higher than average GFR were expected to have lower than average sCr level and higher than average AER level.
- A UACR value established using FMV or 24 h collection samples should in essence be proportional when all else remains identical (e.g. same individual and collection date).
- The effects of ASP8232 were assumed to be reversible.
- ASP8232 was assumed to have no effect on uCr and urine volume.
- The following effects of ASP8232 were considered possible:
- Acute decline of eGFR CysC
- Effect on the chronic eGFR CysC slope
- Reduction of albuminuria (AER and UACR)
- Inhibition of tubular creatinine secretion resulting in increased sCr level

### Model development

A NLME ER model was developed to describe the effect of ASP8232 on GFR, albuminuria and serum creatinine in DKD patients. The dependent variables for the ER analysis were eGFR CysC, sCr, uCr, urine volume, log-transformed AER and log-transformed UACR. Residual variability was evaluated using an additive error model for the log-transformed variables and with a proportional error model for all other variables. Parameter estimation and model evaluation was performed as described in the companion paper [#]. Candidate models were evaluated using the following criteria: plausibility and precision of the parameter estimates, drop in objective function value (OFV), model stability, parameter correlations, and goodness-of-fit plots. For nested models, added parameters were considered significant at p < 0.01, based on a likelihood-ratio test assuming the difference in OFV is χ^2^ distributed (e.g. for 1 additional parameter, a drop in OFV > 6.63 was considered significant). Visual predictive checks (VPC [18, 19]) were performed based on 500 replications of the original dataset and were stratified per renal marker and treatment group.

The baselines of eGFR CysC, sCr, AER, uCr and urine volume were parameterized with a typical value (${\theta }_{TVeGFR}, {\theta }_{TVsCr}, {\theta }_{TVAER}, {\theta }_{TVuCr}$ and ${\theta }_{TVuvol}$) and log-normal distributed inter-individual variability (IIV). The individual (baseline) UACR was calculated from the individual model predicted (baseline) AER (AER_i_ in mg/24 h), uCr (uCr_i_ in g/(L.24 h)) and urine volume (uvol_i_ in L/24 h) using Eq. 1.

1 $$ UACR_{i} \left( t \right) = \theta_{scale} . \frac{{AER_{i} \left( t \right)}}{{uCr _{i} . uvol_{i} }} $$

where UACR_i_ was the individual FMV UACR (mg albumin per g creatinine). The need to add a scaling factor ($\theta$_scale_) was evaluated.

First, a model was developed for the placebo patients in order to characterize the link between the dependent variables, as well as eGFR CysC and AER progression in the absence of ASP8232 treatment (baseline and progression model). Throughout this paper, AER progression refers to increased AER due to incremental damage to the glomerular filtration barrier, while eGFR progression refers to reduced GFR due to parenchymal damage and reduced filtration capacity. A progression component was implemented for eGFR CysC, using an exponential decline with IIV on the rate of decline (Eq. 2). A circadian rhythm was expected [20, 21] and evaluated using a proportional cosine function, with estimated amplitude (with exponential IIV) and time of maximum of the circadian wave (Eq. 3).

2 $$ eGFR_{i} \left( t \right) = \theta_{TVeGFR} \cdot e^{{\eta_{i,1} }} \cdot e^{{ - (\theta_{eGFRt} + \eta_{i,6} ).\frac{t}{10000}}} \cdot CR\left( {tclock} \right) $$

3 $$ CR\left( {tclock} \right) = \left( {1 + \theta_{Ampli} \cdot e^{{\eta_{i,7} }} \cdot {\cos}\left( {2\pi \cdot \frac{{tclock + \left( {24 - \theta_{tmax} } \right)}}{24}} \right)} \right) $$

where eGFR_i_(t) was the model-predicted eGFR CysC for individual i as a function of time after first dose t (h), θ_TVeGFR_ (mL/min/1.73m^2^) was the baseline eGFR CysC (disregarding circadian variation), θ_eGFRt_ (h^−1^) was the rate constant for eGFR CysC progression, θ_Ampli_ and θ_tmax_ (h) were the amplitude and time of maximum of eGFR CysC circadian rhythm, respectively, and tclock (h) represented the clock time. For the i^th^ individual, η_i,1_, η_i,6_ and η_i,7_ were the individual random effects for baseline eGFR CysC, eGFR CysC progression and circadian amplitude, respectively.

The link between eGFR CysC and AER was modeled with a proportional linear function corrected for individual BSA. In addition, a progression component was implemented using an exponential increase with IIV on the rate of increase (Eq. 4).

4 $$ AER_{i} \left( t \right) = \theta_{TVAER} \cdot e^{{\eta_{i,2} }} \cdot e^{{(\theta_{AERt} + \eta_{i,8} ).\frac{t}{10000}}} \cdot \left( {1 + \theta_{AERfiltr} \cdot \left( {eGFR_{i} \left( t \right) \cdot BSA_{i} - \theta_{TVeGFR} \cdot 2.034} \right)} \right) $$

where AER_i_(t) was the model-predicted AER at time t, θ_TVAER_ (mg/24 h) was the baseline AER for an individual with typical baseline eGFR CysC (θ_TVeGFR_) and typical BSA (2.034 m^2^; median value for individuals in dataset), eGFR_i_(t) was given by Eq. 1, θ_AERt_ was the rate constant for AER progression, θ_AERfiltr_ was the slope for the link between filtration and AER. η_i,2_ and η_i,8_ were the individual random effects for baseline AER and AER progression, respectively.

For the link between eGFR CysC and sCr, a proportional linear function as well as a polynomial function (Eq. 5) were evaluated.

5 $$ sCr_{i} \left( t \right) = \frac{{\theta_{TVsCr} }}{{2 - \theta_{sCrfiltr} }} \cdot e^{{\eta_{i,3} }} \cdot \left( {1 - \frac{{\theta_{sCrfiltr} \cdot \left( {\frac{{eGFR_{i} \left( t \right)}}{{\theta_{TVeGFR} }}} \right)^{2} - 1}}{{\frac{{eGFR_{i} \left( t \right)}}{{\theta_{TVeGFR} }}}}} \right) $$

where sCr_i_(t) was the model-predicted sCr at time t, θ_TVsCr_ (µM) was the baseline sCr for an individual with typical baseline eGFR CysC (θ_TVeGFR_), θ_sCrfiltr_ was the parameter of the polynomial function for the link between filtration and sCr. η_i,3_ was the individual random effect for baseline sCr.

uCr was modeled in function of urine volume according to a proportional function (Eqs. 6 and 7).

6 $$ uvol_{i} = \theta_{TVuvol} \cdot e^{{\eta_{i,4} }} $$

7 $$ uCr_{i} = \theta_{TVuCr} \cdot e^{{\eta_{i,5} }} \cdot \left( {1 - \theta_{vol} \left( {uvol_{i} - \theta_{TVuvol} } \right)} \right) $$

where uvol_i_ was the model-predicted urine volume, θ_TVuvol_ (L) was the typical baseline urine volume, uCr_i_ was the model-predicted uCr, θ_TVuCr_ (mM) was the baseline uCr for an individual with typical baseline urine volume (θ_TVuvol_), θ_vol_ was the slope for the link between urine volume and uCr. η_i,4_ and η_i,5_ were the individual random effects for baseline urine volume and uCr, respectively.

Once the best functional forms for the baseline and progression models were identified, data from ASP8232 treated subjects were included to evaluate the pre-specified potential drug effects on eGFR CysC, AER and sCr following an iterative process. Each drug effect was implemented as a direct effect in function of C_u_ by evaluating and comparing different concentration-effect relationships (e.g. additive or proportional; linear, sigmoid emax, logarithmic or exponential) based on the pre-specified criteria.

For each key model and implemented drug effect, it was evaluated whether an improved fit was obtained by replacing C_u_ in the C_u_-effect relationship with VAP-1 plasma activity. If a concentration-effect relationship was not supported by the data, a treatment effect, which was the same for all subjects in the ASP8232 treatment group and would remain for the whole duration of the study including the follow-up visits, was evaluated. Such a treatment effect, whereby the effect persists into the washout phase, was only considered if the data did not support a standard concentration-effect relationship or treatment effect which wears off during washout, obtained by fixing the hill coefficient of a sigmoid emax model to a sufficiently large value. Finally, an indirect-response model was compared to the final direct-effect model, to determine whether a delay between PK and PD could be established. This was performed by using the individual PK parameters obtained from the PK-VAP-1 model described in a companion paper [#], to allow the full PK profile of each individual to drive the response.

Upon inclusion of drug effects and implementation of an adequate individual random effect structure, a covariate analysis was performed to evaluate the possible contribution of covariates to the variability. The statistical covariate analysis was done in a forward addition and backward elimination fashion at significance level of 0.05 and 0.01, respectively. The following covariates were considered: body surface area (BSA, m^2^), sex, age (y), baseline serum albumin (g/L) and DKD disease duration (y). Covariates were evaluated only on parameters with IIV and only if a relationship was apparent based on individual random effect *versus* covariate plots.

### Simulations

Simulations were performed for a typical subject to quantify the relationship between GFR (model predicted eGFR CysC), albuminuria (model predicted AER and UACR) and sCr and the progression of these markers during 1 year in the absence of ASP8232 treatment. Simulations were also performed to quantify the contribution of each ASP8232 effect on GFR, albuminuria and sCr following 40 mg qd ASP8232 for 2 and 12 weeks. A typical subject was a 69 years old male subject with BSA of 2.034 m^2^ and the following baseline characteristics: Serum albumin of 42 g/L, eGFR-CysC of 37.1 mL/min/1.73m^2^, AER of 983 mg/24 h, sCr of 146 uM. C_u_ was simulated using the PK-VAP-1 model described in the companion paper [#], resulting in C_u_ at steady-state of 125.58 nM.

## Results

During exploratory runs, four outliers were excluded from further analysis to stabilize the model based on the criteria defined in the methods section. The link between the dependent variables as well as progression in the absence of ASP8232 treatment was well described by Eqs. 1, 2, 3, 4, 5, 6 and 7. The scale factor, $\theta$_scale_, between AER and UACR in Eq. 1 significantly improved the model fit (ΔOFV =  − 19), and it was therefore kept in the model. The scale factor was estimated to 0.86, suggesting lower albuminuria as measured by FMV UACR *versus* 24 h collection samples. The exponential decay in eGFR CysC and increase in AER could describe the observed progression, and therefore no other functional forms were evaluated. The link between AER and eGFR CysC was well described by a proportional linear function (Eq. 4), whereas the link between sCr and eGFR CysC was best described by a polynomial function (Eq. 5).

Several statistically significant covariate relationships were implemented in the progression model, explaining a part of the inter-individual variability. Baseline eGFR CysC was found to be lower for females (i.e. 83% of the value for males). AER decreased with increasing baseline serum albumin. sCr was found to decrease with age. sCr and uCr were lower for females (i.e. 77% of the value for males), and uCr and urine volume decreased with BSA.

**Fig. 1:**
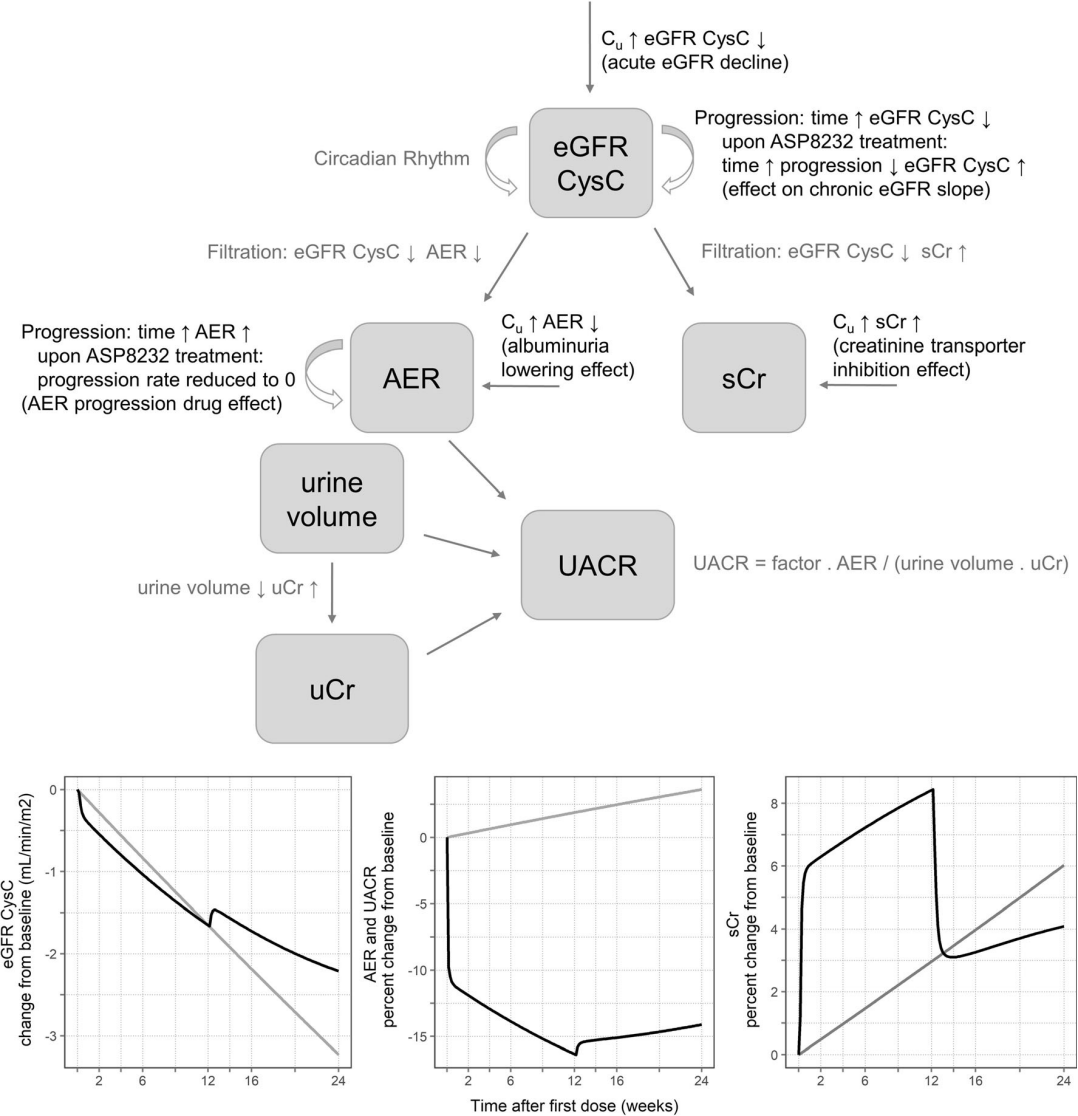
Schematic representation of the ASP8232 Exposure–Response model. The three figures show the model predicted profile for a typical individual in the absence (gray) and presence (black) of 12-week 40 mg qd ASP8232 treatment. *UACR* urinary albumin-to-creatinine ratio, *eGFR CysC* estimated glomerular filtration rate for serum cystatin C, *sCr* serum creatinine, *AER* albumin excretion rate, *uCr* urine creatinine, *C*_*u*_ individual ASP8232 plasma concentrations not bound to VAP-1 (Color figure online)

A schematic overview of the final structural model is presented in Fig. 1. Two ASP8232 drug effects on eGFR CysC were included in the model: an acute eGFR decline of C_u_ on eGFR CysC and an effect of ASP8232 treatment on the chronic eGFR slope (Eq. 8).

8 $$ eGFR_{i,drug} \left( t \right) = \left( {\theta_{TVeGFR} \cdot e^{{\eta_{i,1} }} - \theta_{acute} \cdot C_{u} \left( t \right)} \right) \cdot e^{{ - \left( {\theta_{eGFRt} \cdot \left( {1 - \theta_{chronic} \cdot \frac{t}{10000}} \right) + \eta_{i,6} } \right) \cdot \frac{t}{10000}}} \cdot CR $$

where CR was the eGFR CysC circadian rhythm component as defined in Eq. 3, and eGFR_i,drug_ was the model-predicted eGFR CysC as a function of C_u_ with estimate parameter θ_acute_, and as a function of ASP8232-treatment with estimated parameter θ_chronic_.

Two ASP8232 drug effects were included in the model on AER: an albuminuria lowering effect of ASP8232 on AER according to a sigmoid Imax relationship and an AER progression drug effect whereby the increase in AER was stopped upon ASP8232 treatment, i.e. ${\theta }_{AERt}$ = 0 (Eq. 9):

9 $$ AER_{i,drug} \left( t \right) = AER_{i} \left( {t,\theta_{AERt} = 0} \right) - \frac{{\theta_{Imax} \cdot logC_{u} \left( t \right)^{{\theta_{Hill} }} }}{{\theta_{IC50}^{{\theta_{Hill} }} + logC_{u} \left( t \right)^{{\theta_{Hill} }} }} $$

where AER_i_(t, θ_AERt_ = 0) was the individual AER at time t as described by the progression model (Eq. 4 without progression component; θ_AERt_ = 0: for subjects treated with ASP8232, AER does not increase with time) and AER_i,drug_ was the model-predicted AER as a function of log-transformed C_u_ according to a sigmoid imax relationship with parameters θ_Imax_, θ_IC50_ and θ_Hill_, where the hill coefficient was fixed to 10 (estimating the hill coefficient improved the fit significantly, and resulted in a large and unreliable estimate, and which was arbitrarily fixed to 10). After optimization of the albuminuria lowering effect, it was evaluated whether the data supported a different rate constant for AER progression for ASP8232 treated subjects *versus* placebo (parameter θ_AERt_ in Eq. 4). This was significant and the estimate of θ_AERt_ for ASP8232 treated subjects tended to zero and was subsequently fixed to zero (θ_AERt_ = 0) to improve model stability.

A creatinine transporter inhibition effect of C_u_ on sCr was implemented in the model according to a proportional emax function (Eq. 10)

10 $$ sCr_{i,drug} = sCr_{i} \cdot \left( {1 + \frac{{\theta_{Emax} \cdot e^{{\eta_{i,9} }} \cdot C_{u} }}{{\theta_{EC50} + C_{u} }}} \right) $$

where sCr_i,drug_ was the model-predicted sCr as a function of C_u_ according to an emax relationship with estimated parameters θ_Emax_ and θ_EC50_ (nM) and sCr_i_ was the model-predicted sCr in the absence of drug as specified in Eq. 5. η_i,9_ was the individual random effect for Emax of the creatinine transporter inhibition effect. It was assumed that the total amount of creatinine in the urine reflects the total amount of produced creatinine in the body. Creatinine production is anticipated to be independent of creatinine transporter inhibition. Therefore, the creatinine transporter inhibition effect was implemented on sCr, but not on uCr.

An indirect response model did not improve the fit significantly (p > 0.01), and therefore the direct effect model was selected as the final model.

### Model validation

The parameter estimates and precision of the estimates are presented in Tables 1 and 2. The NONMEM model code can be found in the Supplementary Material. All effects included in the model were significant at p < 0.01, based on likelihood-ratio tests. Most parameters were estimated with acceptable precision, although for some parameters the relative standard error (RSE, %) was high, mainly for the slope of the acute eGFR decline (63%), the effect on the chronic eGFR slope (49%) and the amplitude for eGFR CysC circadian variation (46%). The eta shrinkage was 36% for IIV on AER progression and 39% for IIV on the emax of the creatinine transporter inhibition effect, and less than 20% for other parameters. The epsilon shrinkage was less than 15% for all variables. Correlations between structural parameter estimates lied between − 0.9 and 0.9, with the strongest correlation, − 0.9, observed between the baseline eGFR CysC and the baseline sCr.

**Table 1:** Structural effect parameters of the ASP8232 Exposure–Response model

Structural effect parameter (unit)	Implementation	Value	RSE (%)	θ Nr
Baseline eGFR CysC (mL/min/1.73m^2^)		37.1	4.2	θ_2_
eGFR CysC progression	. e^−θ3.TAFD/10000^	0.226	18	θ_3_
Slope of acute eGFR decline	− θ_4_. C_u_	0.00218	63	θ_4_
Chronic eGFR slope effect	. (1–θ_5_. TAFD/10,000)	0.807	49	θ_5_
Baseline AER (mg/24 h)		983	9.6	θ_7_
AER filtration	. (1 + θ_8_. (eGFR_i_. BSA–θ_2_ .2.034))	0.0196	7.7	θ_8_
Imax of albuminuria lowering effect	− θ_9_. logC_u_^θ24^/(θ_23_^θ24^ + logC_u_^θ24^)	95.9	39	θ_9_
Baseline sCr (µM)		146	2.9	θ_11_
sCr filtration	. (1–θ_12_. (eGFR_i_ /θ_2_)^2^–1)/(eGFR_i_ /θ_2_)	0.171	19	θ_12_
Emax of creatinine transporter inhibition	. (1 + θ_13_. C_u_/ (θ_31_ + C_u_))	0.0753	17	θ_13_
Baseline urine volume (L)		2.01	2.7	θ_15_
Baseline uCr (mM)		5.30	3.2	θ_17_
Urine volume—uCr link function	. (1–θ_18_. (uvol_i_–θ_15_))	0.304	13	θ_18_
UACR scale factor	UACR_i_ = θ_20_. (AER_i_/(uvol_i_.uCr_i_)	0.858	2.5	θ_20_
AER progression	. e^θ21.TAFD/10000^	0.430	38	θ_21_
AER progression drug effect	. e^θ22.TAFD/10000^	0^a^	–	θ_22_
IC_50_ of albuminuria lowering effect	− θ_9_. logC_u_^θ24^/(θ_23_^θ24^ + logC_u_^θ24^)	5.95	4.7	θ_23_
Hill coefficient of albuminuria lowering effect	− θ_9_. logC_u_^θ24^/(θ_23_^θ24^ + logC_u_^θ24^)	10.0^a^	–	θ_24_
Sex covariate effect on sCr and uCr		0.770	2.4	θ_25_
Age covariate effect on sCr		− 0.595	17	θ_26_
Baseline albumin covariate effect on AER		− 3.86	22	θ_27_
Sex covariate effect on eGFR CysC		0.829	5.3	θ_28_
BSA covariate effect on urine volume		0.732	41	θ_29_
BSA covariate effect on uCr		1.21	19	θ_30_
EC_50_ of creatinine transporter inhibition (nM)	. (1 + θ_13_. C_u_/(θ_31_ + C_u_))	52.9^a^	–	θ_31_
Amplitude of eGFR CysC circadian rhythm	. (1 + θ_32_. cos(2π. (clocktime + (24-θ_33_))/24))	0.0783	46	θ_32_
Maximum of eGFR CysC circadian wave (h)	. (1 + θ_32_. cos(2π. (clocktime + (24-θ_33_))/24))	10.5	15	θ_33_

^a^Fixed; *RSE* relative standard error, *θ Nr* THETA number in NONMEM code (Supplementary Material), *C*_*u*_ model-predicted unbound ASP8232 plasma concentration in nM, *logC*_*u*_ log(1 + 1000. C_u_), *TAFD* time after first dose, *BSA* body surface area, *UACR* urinary albumin-to-creatinine ratio, *eGFR CysC* estimated glomerular filtration rate for serum cystatin C, *sCr* serum creatinine, *AER* albumin excretion rate, *uCr* urine creatinine

**Table 2:** Random effect parameters of the ASP8232 exposure–response model

Random effect parameter	Value	RSE (%)
ω^2^_Baseline eGFR CysC_	0.0816	14
ω _Baseline eGFR CysC, AER_	− 0.112	26
ω^2^_Baseline AER_	0.523	17
ω^2^_Baseline sCr_	0.0133	13
ω^2^_Baseline urine volume_	0.0720	23
ω^2^_Baseline uCr_	0.0479	17
ω^2^_eGFR CysC progression_	0.0559	22
ω^2^_Circadian amplitude_	1.18	57
ω^2^_AER progression_	1.01	23
ω^2^_Creatinine transporter inhibition_	0.467	43
σ _eGFR CysC,proportional_	0.0987	3.2
σ _log(AER),additive_	0.424	6.1
σ _sCr,proportional_	0.0857	3.9
σ _urine volume,proportional_	0.165	5.2
σ _uCr,proportional_	0.304	4.6
σ _log(UACR),additive_	0.342	4.1

*ω*^*2*^ variance of inter-individual variability, *σ* standard deviation of residual error

**Fig. 2:**
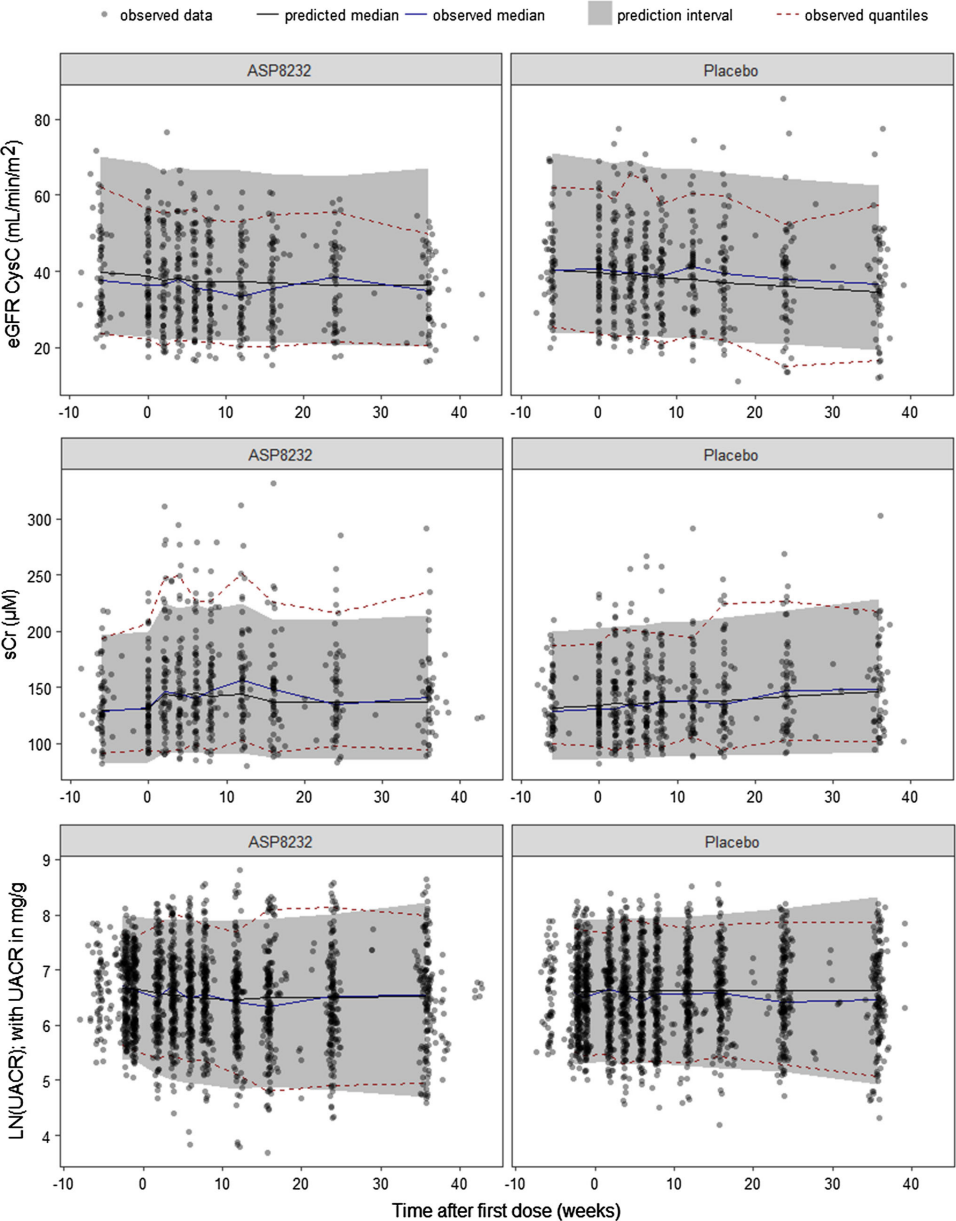
Visual predictive check of eGFR CysC, sCr and UACR. The observed data (dots), median (blue line), 5th and 95th percentiles (dashed red lines), and the predicted median (black line) and 90% prediction interval (grey area) are shown. *UACR* urinary albumin-to-creatinine ratio, *eGFR CysC* estimated glomerular filtration rate for serum cystatin C, *sCr* serum creatinine (Color figure online)

**Fig. 3:**
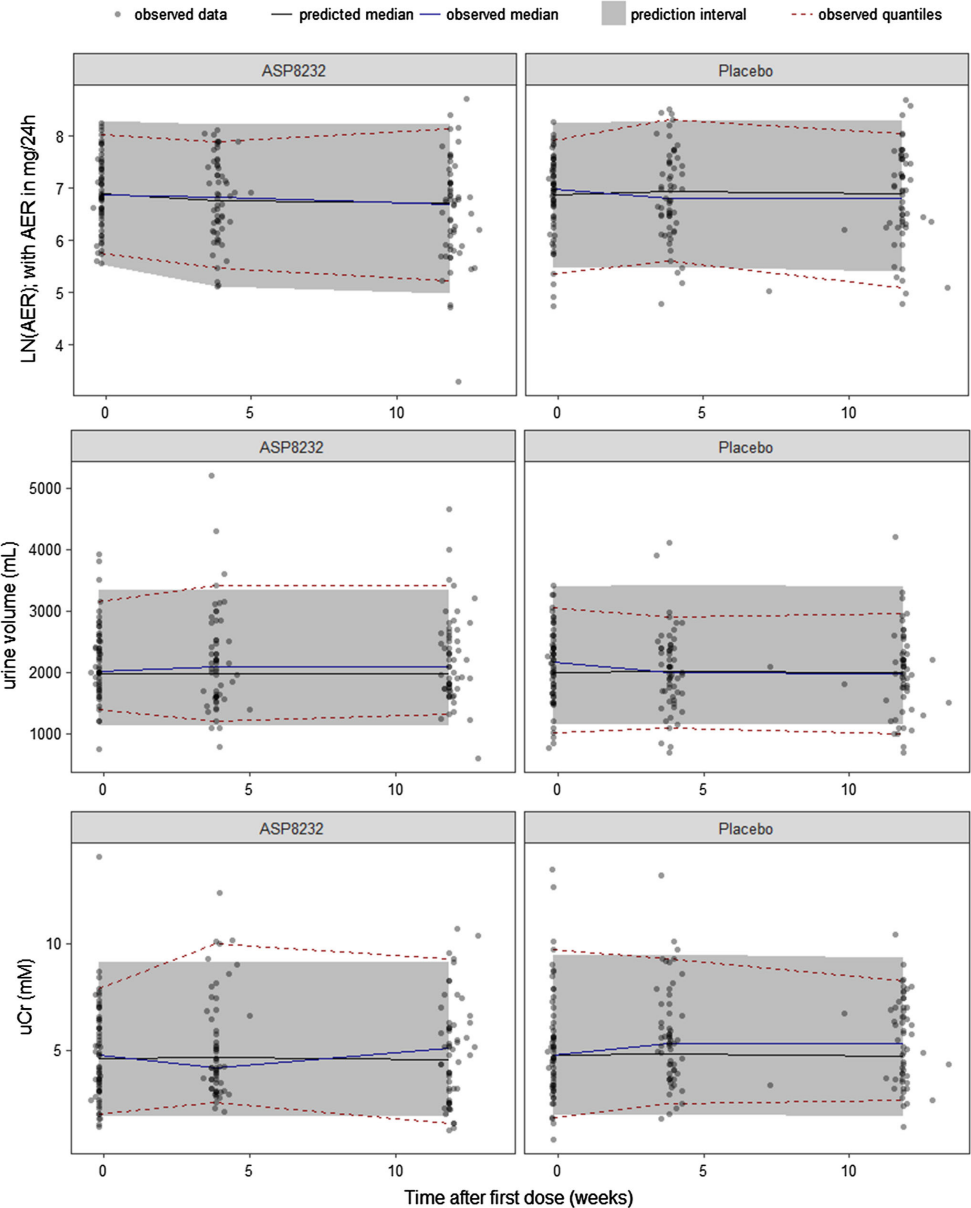
Visual predictive check of AER, urine volume and uCr. The observed data (dots), median (blue line), 5th and 95th percentiles (dashed red lines), and the predicted median (black line) and 90% interval (area) of the 5th and 95th predicted percentiles are shown. *AER* albumin excretion rate, *uCr* urine creatinine (Color figure online)

**Fig. 4:**
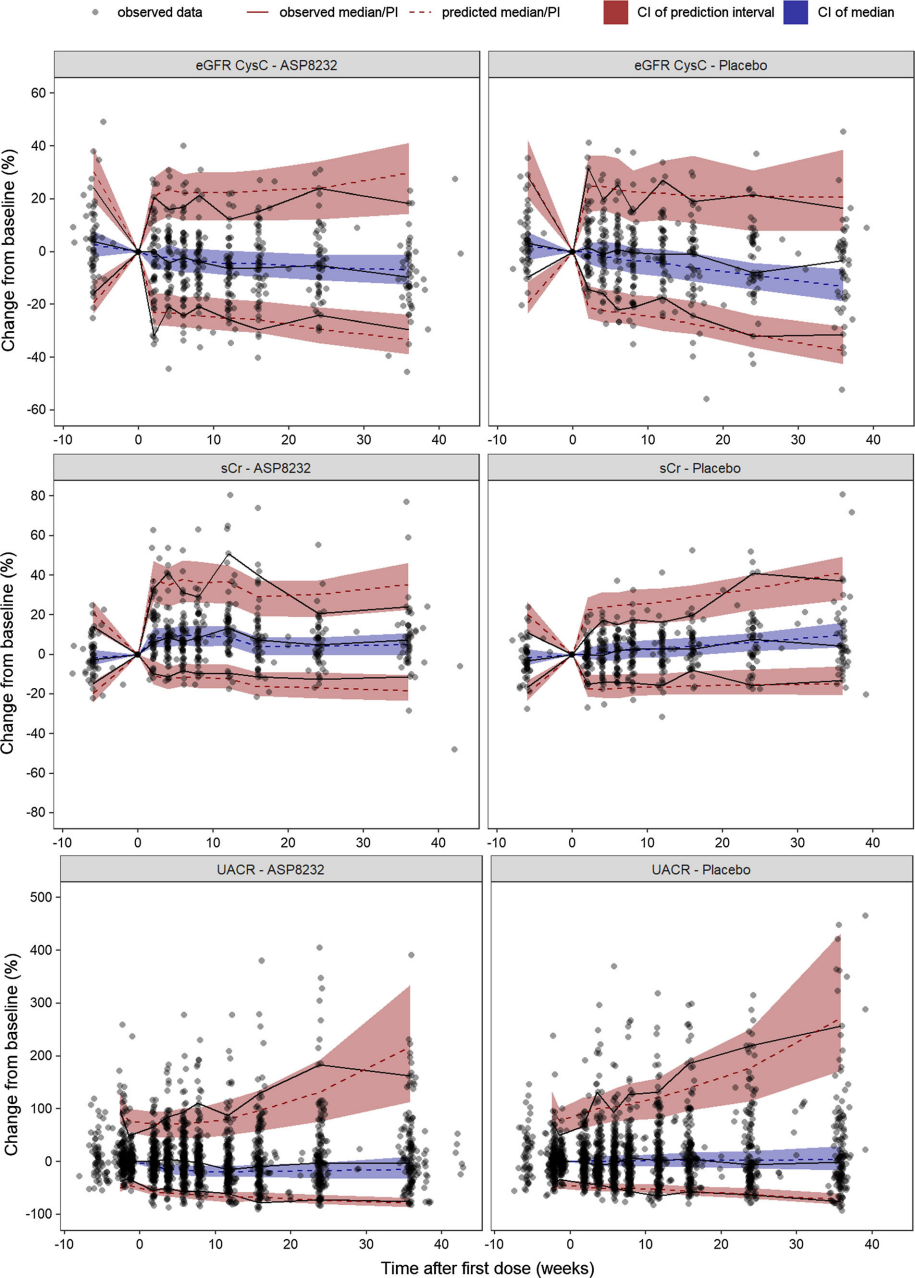
Percent change from baseline visual predictive check of eGFR CysC, sCr and UACR. The observed observations (dots), observed median, 5th and 95th predicted percentiles (solid lines), predicted median, 5th and 95th predicted percentiles (dashed lines) and their 95% confidence intervals (area) are shown. *PI* prediction interval, *CI* confidence interval, *UACR* urinary albumin-to-creatinine ratio, *eGFR CysC* estimated glomerular filtration rate for serum cystatin C, *sCr* serum creatinine. Individuals with missing baseline (defined as up to 2 weeks before the first dose) are not included. For UACR, the median of the observations and predictions at baseline per individual was used to generate change from baseline values. For clarity, five UACR change from baseline observations that exceeded 500% were excluded

**Fig. 5:**
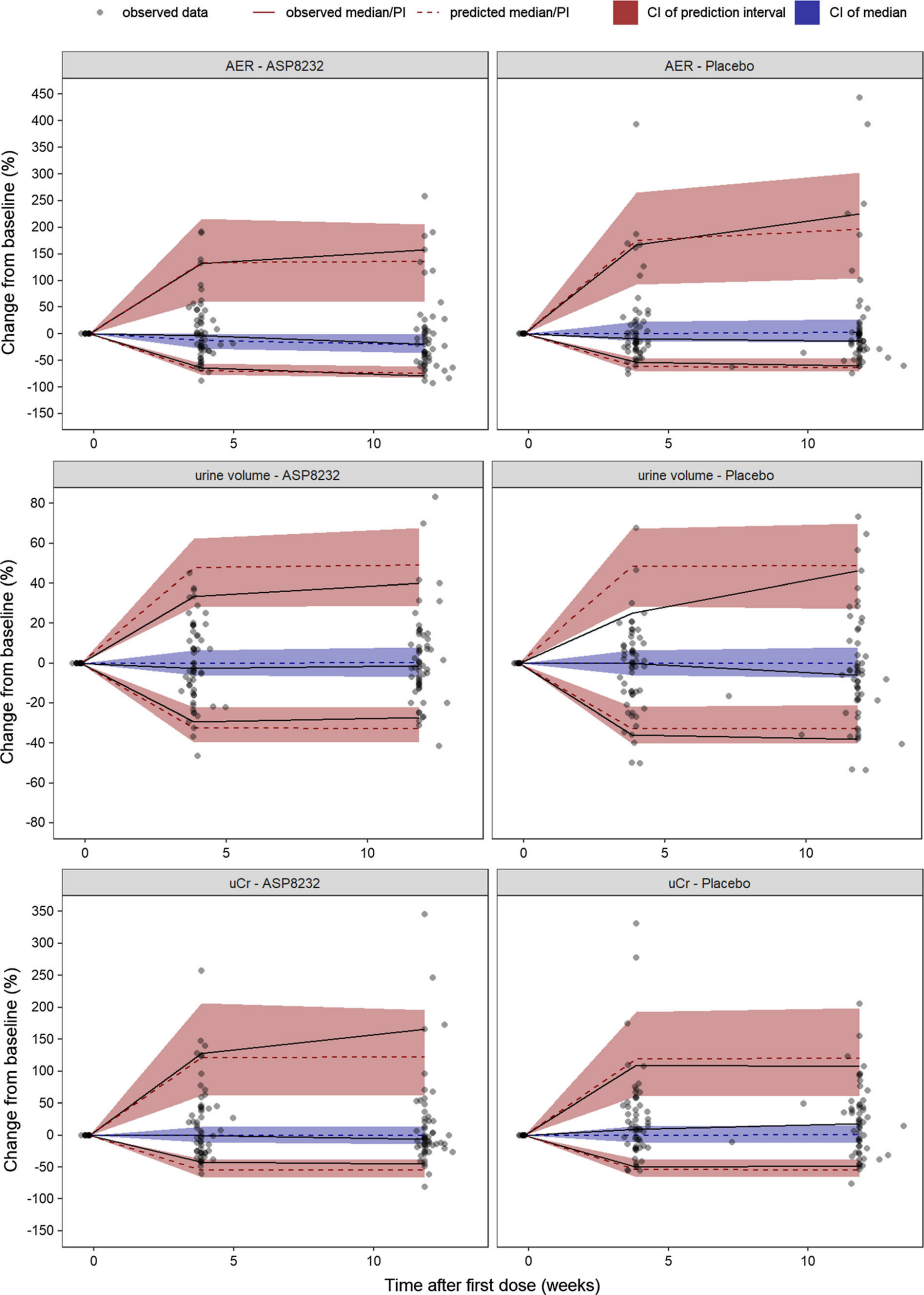
Percent change from baseline visual predictive check of AER, urine volume and uCr. The observed observations (dots), observed median, 5th and 95^th^ predicted percentiles (solid lines), predicted median, 5th and 95th predicted percentiles (dashed lines) and their 95% confidence intervals (area) are shown. *PI* prediction interval; *CI* confidence interval, *AER* albumin excretion rate, *uCr* urine creatinine. Individuals with missing baseline (defined as up to 2 weeks before the first dose) are not included

The VPC’s (dependent variable–based and percent change from baseline–based) are presented in Figs. 2 and 4 for eGFR CysC, sCr and UACR, and in Figs. 3 and 5 for AER, uCr and urine volume. Overall, it can be concluded that the ASP8232 model is able to adequately describe the central tendency over time and variability in the data from the Phase 2 study in DKD patients. A small decline in eGFR CysC following treatment was observed and attributed to a potential acute and reversible hemodynamic effect of ASP8232 [3]. For eGFR CysC, the model captures this observed small drop after the first month of treatment (Fig. 4). eGFR declines with time in the placebo group, and the model predicts a slower eGFR decline for ASP8232-treated patients (Figs. 2 and 4). The model appears to slightly over-predict the variability for higher eGFR CysC values. The model can describe the increase in sCr with time, which is especially apparent in placebo patients. In the model, this increase in sCr is driven by the decrease in eGFR. A slight under-prediction of the variability for higher sCr values is observed. The increase in sCr upon ASP8232-treatment is clearly well-captured by the model (Figs. 2 and 4).

The model can adequately describe the UACR and AER lowering effect of the compound following ASP8232 treatment (Figs. 2, 3, 4 and 5), without the need to estimate a different individual baseline or drug effect for UACR as compared to AER. This supports the hypotheses that UACR measurements using FMV and 24 h urine collections are proportional. However, the central tendency for UACR appears over-predicted during the first weeks of treatment (Fig. 4), suggesting that the exposure–response relationship might be more complex than currently supported by the available data.

For AER, uCr and urine volume, 24 h collections only occurred at 3 time-points in the study (at week 0, 4 and 12). Thus, the VPC’s for these variables are less informative, although they do show an adequate description of the variability and median trend in the data for these variables (Figs. 3 and 5).

**Fig. 6:**
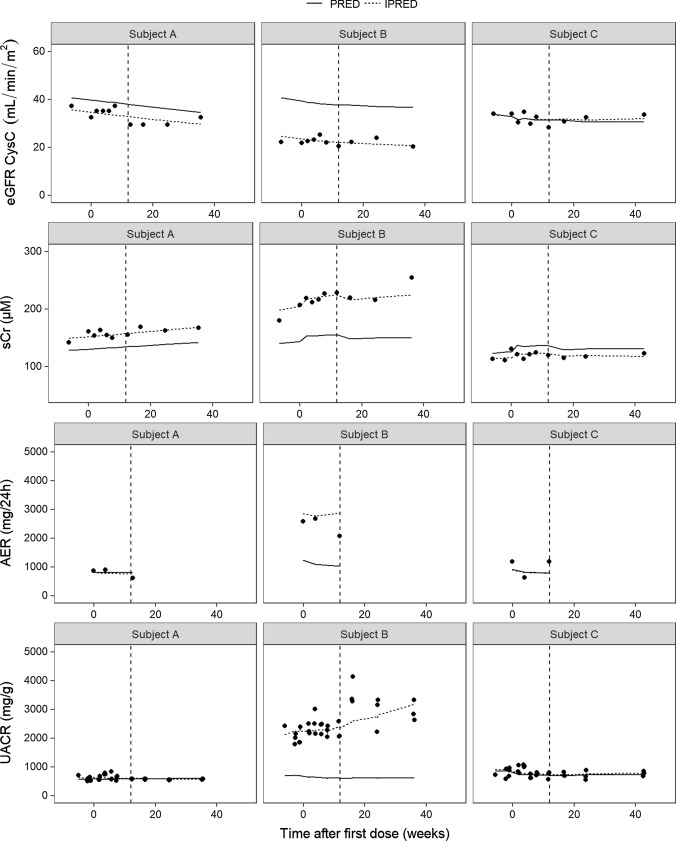
Individual fits for eGFR CysC, sCr, AER and UACR of selected subjects. Observations (dots), population predictions (PRED; solid line) and individual predictions (IPRED; dashed line) are shown versus time after first dose. *UACR* urinary albumin-to-creatinine ratio, *eGFR CysC* estimated glomerular filtration rate for serum cystatin C, *sCr* serum creatinine, *AER* albumin excretion rate

To further illustrate the ability of the model to describe the data for all observed variables simultaneously, individual fits are presented for three selected subjects (Fig. 6). These subjects were selected arbitrarily, solely to further explain the model structure on an individual level. Subject A received placebo. Measured eGFR CysC levels were lower than average for this subject, leading to subsequently higher than average sCr levels. With time, eGFR CysC declined and sCr increased. AER levels were close to the population value for this subject, and thus the same was observed for UACR levels.

Subject B was treated with ASP8232 for 12 weeks. The creatinine transporter inhibition effect can clearly be seen for this individual, as sCr concentrations are elevated during treatment. This effect appears to be reversible as sCr values decreased at end-of-treatment (week 12). Such a change during the treatment phase was not observed for eGFR CysC for this subject, consistent with an effect on creatinine transporters. In addition, higher than average AER and UACR values were observed for this individual. The albuminuria lowering effect during treatment is apparent from the UACR data and captured well by the model. There is a less pronounced increase in albuminuria during treatment as compared to after treatment. The AER data, with only 3 available measurements were less informative. Due to the simultaneous nature of the model, the UACR data was able to inform the most likely albuminuria level for subject B, hereby the individual AER prediction appears to follow the UACR trend in the data more than the AER trend.

For ASP8232-treated Subject C, the values for all variables are close to the population. For this subject, the model-predicted acute decline in eGFR CysC can be observed upon initiation of treatment, while the creatinine transporter inhibition effect is clearly visible in the sCr data and the albuminuria lowering effect can be observed in the UACR FMV data. Figure 6 shows that through integration of all data, the model can capture all these above-mentioned trends.

### Simulations

The progression model predicts that for a typical placebo subject, after 1 year, albuminuria would increase by 7%, and the sCr-based eGFR using the CKD-EPI equation [22] would decrease with 6 mL/min/1.73m^2^, which is in line with expectations.

**Fig. 7:**
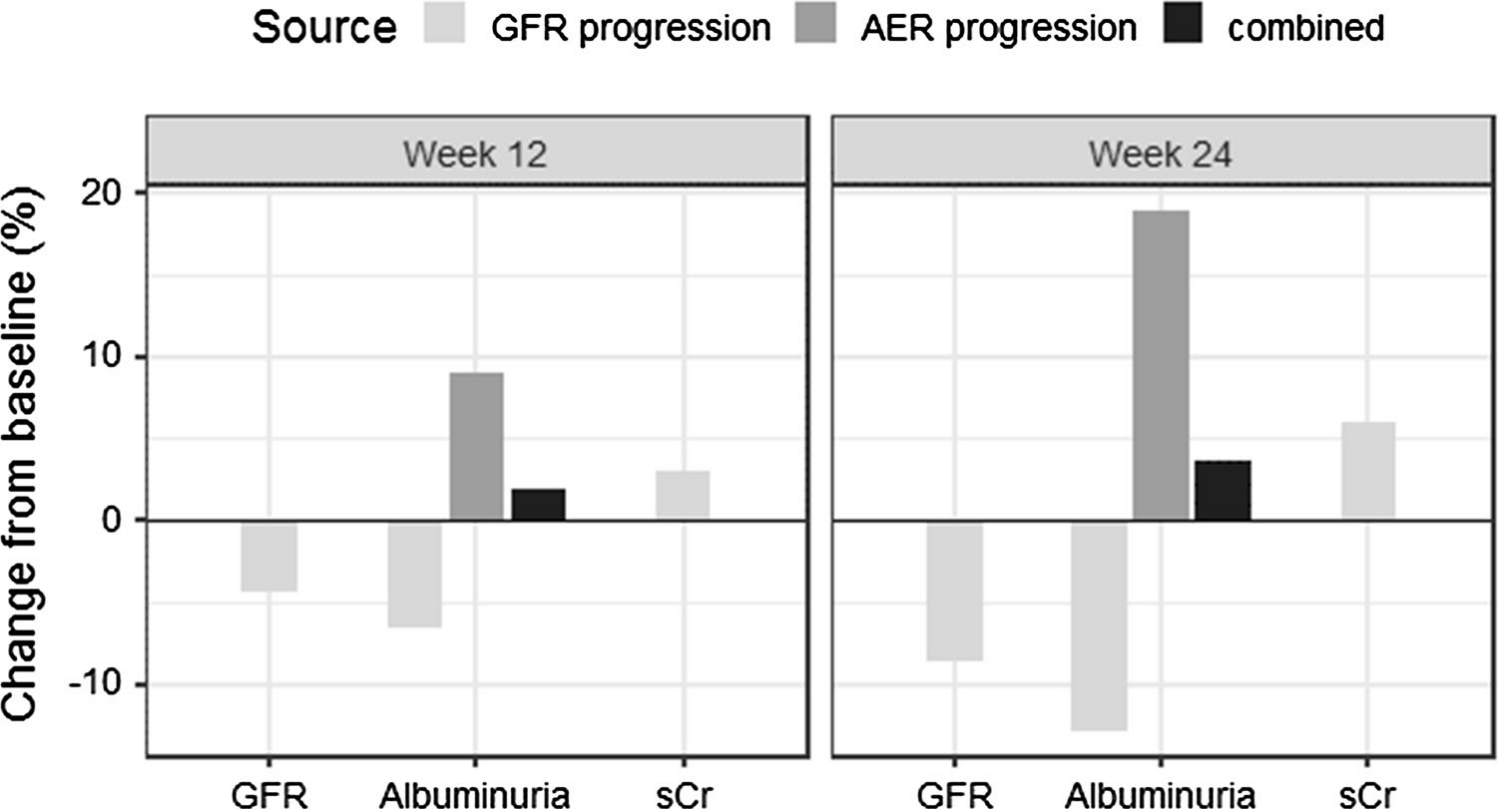
Quantification of progression for a typical subject in absence of ASP8232 treatment. *GFR* glomerular filtration rate, *sCr* serum creatinine, *AER* albumin excretion rate

At week 12 and 24, the progression model predicts a 4.4% and 8.7% reduction in GFR for a typical placebo subject as compared to baseline, causing a 6.6% and 12.9% reduction in albuminuria, respectively (Fig. 7). The model predicts that this effect is countered by an albuminuria progression of 9.1% and 18.9%, respectively, resulting in an overall increase in albuminuria with 1.9% and 3.6% after 12 and 24 weeks, respectively.

**Fig. 8:**
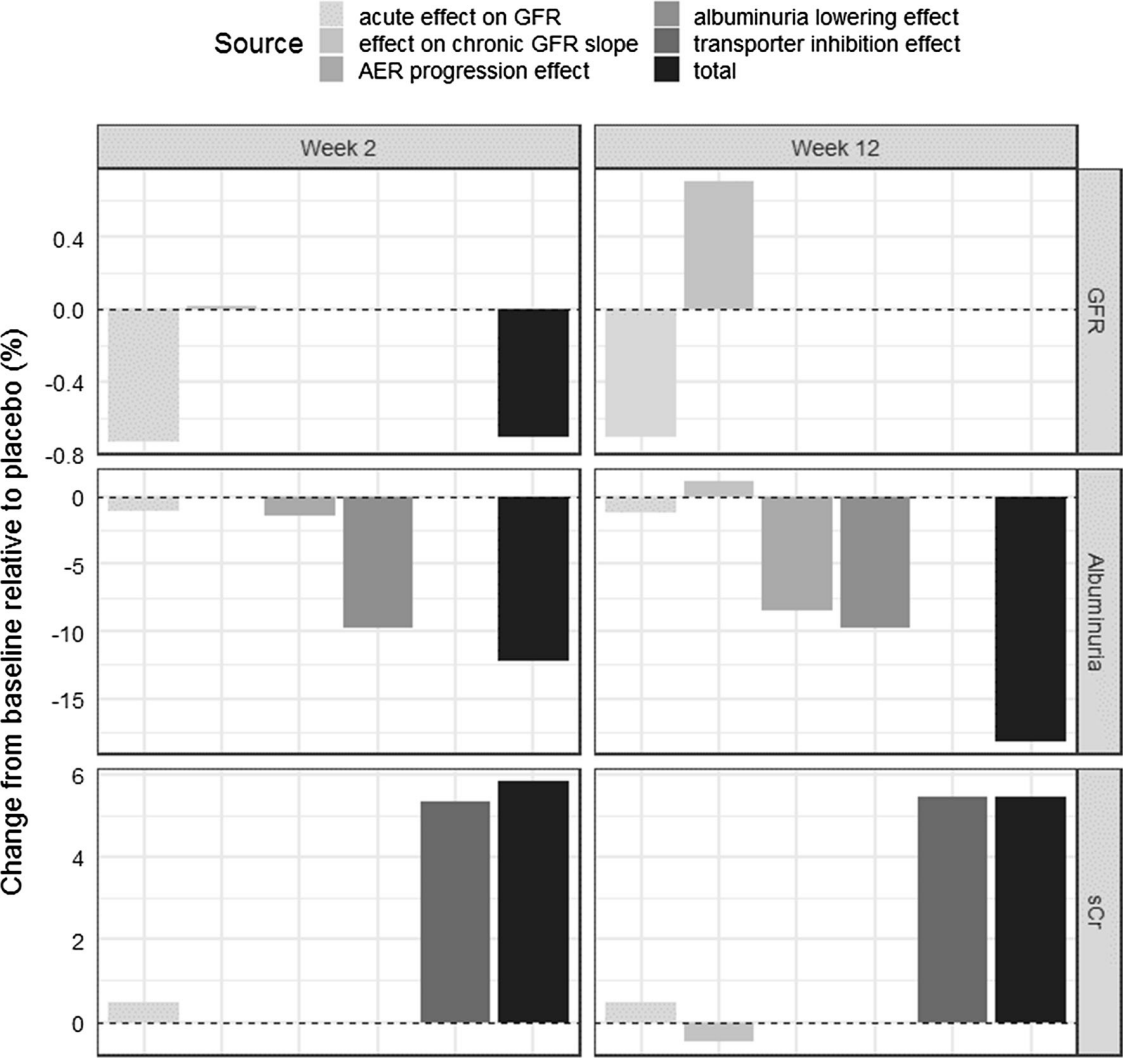
Quantification of progression for a typical subject following ASP8232 treatment. The bars show the percent change from baseline caused by each direct or indirect effect of ASP8232 treatment on GFR, albuminuria and sCr subtracted by the corresponding overall percent change from baseline in an untreated typical subject due to progression. *GFR* glomerular filtration rate, *sCr* serum creatinine, *AER* albumin excretion rate

The placebo-corrected percent change from baseline caused by each direct and indirect ASP8232 effect is presented in Fig. 8. At week 2, the acute GFR effect reduces GFR by 0.73% whereas there is almost no increase caused by the effect on the chronic slope (Fig. 8 top-left). The reduction in GFR causes a decrease in albuminuria by 1.1% (Fig. 8 middle-left) and a 0.47% increase in sCr (Fig. 8 bottom-left). The opposing acute effect and effect on the chronic GFR slope are equal following 12 weeks.

The model contains four different direct or indirect effects of ASP8232 on albuminuria (Fig. 8 middle). At week 2, the acute GFR effect is responsible for only a small part of the total drug effect on albuminuria, which is mainly caused by the albuminuria lowering effect. The removal of the albuminuria progression by ASP8232 treatment results in a 1.4% and 8.5% reduction in albuminuria after 2 and 12 weeks of treatment, respectively.

The inhibition of transporters involved in sCr secretion is the main cause of the increase in sCr as compared to the effect of ASP8232 on GFR (Fig. 8 bottom).

## Discussion

In this NLME ER analysis for a Phase 2 trial in DKD patients (ALBUM study; [3]) where 12-week 40 mg qd ASP8232 treatment demonstrated an albuminuria lowering effect, we have integrated data from all available renal filtration markers and albuminuria assessments. As such, a mechanism-based model was obtained that allows simulations of longitudinal changes at the individual level in markers of progressive DKD, i.e. eGFR and albuminuria, including the treatment effect of ASP8232.

In the absence of ASP8232 treatment, this mechanism-based model could (i) distinguish between the effect of GFR and progression for albuminuria, (ii) link sCr and CysC information to GFR simultaneously and (iii) link 24 h AER and FMV UACR information. Increased AER is caused by incremental damage to the glomerular filtration barrier (i.e. AER progression), while reduced GFR is a result of parenchymal damage and reduced filtration capacity (i.e. eGFR progression).

As expected, a subject with higher than average GFR was found to have lower than average sCr levels and higher than average AER levels in line with the pre-specified model assumptions. The relationship between eGFR CysC and sCr was parameterized with a proportional function, which described not only the relationships between subjects at baseline but also the effect of GFR on sCr over time within one subject. Due to progression of disease, eGFR CysC declined with time according to an exponential function, which could explain the observed increase in sCr with time. The model was able to adequately quantify and describe these anticipated drug-independent effects.

The eGFR CysC-AER relationship was parameterized with a proportional linear function by which a decline in eGFR CysC would lead to a decrease in AER. The relationship was assumed to be valid both between subjects and within a single subject over time. This was further supported by a correlation between baseline serum albumin and AER identified during the covariate analysis, with lower serum albumin levels corresponding with higher AER levels. Lower serum albumin levels might result from a higher AER due to structural damages in the glomerular filtration barrier. To account for an overall increase in albuminuria as the disease progresses, an AER progression component was implemented in the model whereby the contribution of each effect could be quantified (Fig. 7).

We evaluated four potential effects of ASP8232 treatment: an acute decline of eGFR CysC, an effect on the chronic eGFR CysC slope, a reduction of albuminuria, and an inhibition of tubular creatinine secretion resulting in increased sCr levels. The albuminuria lowering effect was best described by a sigmoid imax model using log-transformed C_u_ as driver of the effect and with fixed hill coefficient to 10. The high hill coefficient indicates that this is mainly a treatment effect. It is likely that this apparent on–off effect is the result of a narrow range of ASP8232 exposures during the treatment period as just one dose was included in the study. Potentially, the albuminuria lowering effect is truly a function of VAP-1 inhibition, but this implementation was inferior, likely due to the nearly complete VAP-1 inhibition during treatment for all subjects. In addition, the data supported a difference in AER progression between treatment groups, which was implemented as a cease of AER progression upon ASP8232 treatment. This allowed an optimal description of AER and UACR (Figs. 2, 3 and 4) and was thus in line with the endpoint analysis in the clinical study. However, the implementation of both the albuminuria lowering effect and the effect on AER progression is mainly empiric. As the GFR and albuminuria effects on AER are in opposite direction resulting in a lower net effect, the confidence of the resulting size of each effect following ASP8232 treatment is low.

Following the inclusion of the anticipated albuminuria and creatinine effects, the potential acute and chronic effects of ASP8232 on eGFR CysC were re-evaluated. An acute and reversible decline of eGFR CysC was observed during ASP8232 treatment [3]. This effect could be implemented in the ER model, as a linear effect in function of C_u_. Although the inclusion of this effect was significant, the slope was estimated with low precision (63% RSE) and its impact on eGFR CysC is predicted to be very small (drop of less than 1%, on average). This is a consequence of integration of all information on filtration and the inclusion of additional model components such as circadian variation, combined with the pre-specified model assumptions. If our model assumptions are correct, it becomes questionable whether the acute effect is a true ASP8232 effect to be expected to re-occur in future trials, or a coincidental artefact of the evaluated Phase 2 study. The hypothesis of this acute effect being an artefact is supported by data from a Phase 2 study in patients with diabetic macular edema (ClinicalTrials.gov NCT02302079), where the same dose level of ASP8232 was evaluated and where an acute eGFR decline was not observed.

Interestingly, the model fit was improved significantly when the exponential decline in eGFR CysC was less for ASP8232 treated subjects. This effect was implemented as a treatment group effect (only for ASP8232 subjects) which started upon treatment and remained during follow-up. When excluding this effect from the model, the eGFR CysC of a typical ASP8232-treated subject was predicted to be 0.7% lower, after 12 weeks of treatment. However, the parameter for this effect was estimated with low precision (49% RSE) and implementation of IIV was not supported. Considering the limitations regarding its implementation, the short duration of treatment of the available data, and low precision of the estimate, the effect on the chronic eGFR slope in the ER model should not be considered as proof of long-term renoprotective effect, but rather as a promising hypothesis to be explored in future studies.

The creatinine transporter inhibition effect was best described as an emax function, with EC_50_ fixed to 52.9 nM, a value obtained from a model incorporating ASP8232 data from 4 clinical trials covering a broad dose range (data on file). This implementation was preferred over a linear effect, which would be the most parsimonious model supported by the data from the ALBUM study only, but would not be mechanistically plausible as a maximal effect is expected at 100% inhibition of secretion transporters. To cover uncertainties presented by fixing the EC_50_, a sensitivity analysis was performed whereby this EC_50_ was increased or decreased with 50% to values of 79.4 or 26.5 nM leading to a change in OFV of 2.1 or − 2.6, respectively. As expected, the impact was largest on the emax parameter of the creatinine transporter inhibition effect: a 9% increase or 10% decrease was observed, respectively. Minor changes were observed for (i) the variance estimate of this emax parameter, (ii) the slope of the acute eGFR decline and (iii) the effect on chronic eGFR slope parameter, where changes between -4 to 6% were observed upon varying the EC_50_. All other parameter estimates either did not change or changed with less than 1%. Overall, it was concluded that fixing the EC_50_ of the creatinine transporter inhibition effect was justifiable.

As shown in Fig. 8, the observed increase in sCr is driven almost entirely by the inhibition of the renal creatinine transporters. As a result, sCr-based eGFR is expected to drop upon treatment almost entirely independent of a change in filtration. Indeed, in the phase 2 study, the most frequently reported drug-related treatment emergent adverse events were renal impairment and decreased glomerular filtration [3]. The model supports that these adverse events are largely linked to a creatinine transporter effect.

The developed ER model was applied to investigate four potential effects of ASP82332 treatment for the ALBUM study. Assumptions and limitations should be taken into consideration when using the model in new situations. Several limitations of the ER model were mentioned and are re-iterated. Since data were available for not more than one ASP8232 dose level (40 mg qd), the AER progression effect and the effect on the chronic eGFR slope were implemented as ASP8232 treatment effects, unrelated to changes in PK or PD. The other ASP8232 drug effects (albuminuria lowering effect, acute eGFR decline and creatinine transporter effect) were implemented as a function of C_u_, whereby inhibition of VAP-1 activity was not the main driver of the effect. Especially for the albuminuria lowering effect, it would be anticipated that this is driven by the PD effect of ASP8232, i.e. VAP-1 inhibition, rather than ASP8232 concentration. Further refinement may be possible when additional clinical data become available. Therefore, it is advised to not extrapolate the drug effects beyond the currently evaluated Phase 2 design, in terms of study duration and dosing. Further, a delay between drug exposure and biomarker response was expected but not significant as tested via indirect response modeling. The reason that these delays could not be quantified might be due to (i) a too sparse sampling schedule, (ii) the actual delays being relatively short, (iii) model limitations due to increasing model complexity.

Within the boundaries of the evaluated conditions, the ER model proved useful to unravel, characterize and quantify the different effects that influence GFR, albuminuria and sCr. The implementation of most drug effects was empirical and would need to be confirmed and refined with additional ASP8232 clinical data. However, the developed progression model is drug-independent and was set up in such a way as to connect all markers in a potentially biologically plausible manner. Thus, this progression model is expected to be applicable as a starting point for other compounds affecting such biomarkers in a similar time scale.

In conclusion, a mechanism-based ER model was successfully developed and used to quantify the relationship and longitudinal association between GFR and albuminuria, uncouple the GFR mediated and direct ASP8232 effects on albuminuria and uncouple the effects of change in GFR and ASP8232 transporter inhibition on sCr. The model suggests that the impact of the observed acute and reversible eGFR CysC decline upon ASP8232 treatment is limited. Although not evident when evaluating each marker separately, early signs of a beneficial effect of ASP8232 on the chronic eGFR slope could be quantified by the ER model by integrating all available information on renal filtration. The model allows quantification and simulations of the longitudinal changes in efficacy assessments related to clinical outcomes in DKD and may therefore be useful for other compounds affecting the same markers in a similar time scale.

## Electronic supplementary material

Below is the link to the electronic supplementary material.

Supplementary file1 (TXT 5 kb)

## Abbreviations

AER: Albumin excretion rate
ALBUM: ASP8232 phase 2 trial in DKD patients: ClinicalTrials.gov NCT02358096
BSA: Body surface area
CI: Confidence interval
CR: EGFR CysC circadian rhythm component
C_u_: Individual ASP8232 plasma concentrations not bound to VAP-1
DKD: Diabetic kidney disease
eGFR: Estimated glomerular filtration rate
ER: Exposure–response
FMV: First morning void
IIV: Inter-individual variability
NLME: Non-linear mixed effects
OFV: Objective function value
PI: Prediction interval
PK-PD: Pharmacokinetic-pharmacodynamic
Qd: Once daily dosing
RSE: Relative standard error
sCr: Serum creatinine
sCysC: Serum cystatin C
T, TAFD: Time after first dose in hours
Tclock: Clock time
TV: Typical value
UACR: Urinary albumin-to-creatinine ratio
uCr: Urine creatinine
Uvol: Urine volume
VAP-1: Vascular adhesion protein-1
VPC: Visual predictive check
η: Random effect for inter-individual variability
θ: Fixed effect model parameter

## List of PK model parameters (THETA number, θ_n_, reflects the number of the parameters in Table 1 and in the NONMEM code (Supplementary Material)

θ_scale_ (θ_20_): Scaling factor between FMV and 24 h urine measurements
θ_TVeGFR_ (θ_2_): Baseline eGFR CysC (disregarding circadian variation)
θ_eGFRt_ (θ_3_): Rate constant for eGFR CysC progression
θ_Ampli_ (θ_32_): Amplitude eGFR CysC circadian rhythm
θ_tmax_ (θ_33_): Time of maximum of eGFR CysC circadian rhythm
θ_TVAER_ (θ_7_): Baseline AER for an individual with typical baseline eGFR CysC and BSA
θ_AERt_ (θ_21_): Rate constant for AER progression
θ_AERfiltr_ (θ_8_): Slope for the link between filtration and AER
θ_TVsCr_ (θ_11_): Baseline sCr for an individual with typical baseline eGFR CysC
θ_sCrfiltr_ (θ_12_): Parameter of the polynomial function for the link between filtration and sCr
θ_TVuvol_ (θ_15_): Typical baseline urine volume
θ_TVuCr_ (θ_17_): Baseline uCr for an individual with typical baseline urine volume
θ_vol_ (θ_18_): Slope for the link between urine volume and uCr
θ_acute_ (θ_4_): Parameter for acute drug effect on eGFR CysC as function of C_u_
θ_chronic_ (θ_5_): Parameter for chronic drug effect on eGFR CysC as function of treatment
θ_Imax_ (θ_9_): Maximum effect of AER imax relationship as function of log-transformed C_u_
θ_IC50_ (θ_23_): Log-transformed C_u_ to reach half maximal effect of AER imax relationship
θ_Hill_ (θ_24_): Hill coefficient of AER imax relationship as function of log-transformed C_u_
θ_Emax_ (θ_13_): Maximum effect of sCr emax relationship as function of C_u_
θ_EC50_ (θ_31_): C_u_ to reach half maximal effect of sCr emax relationship
ω^2^: Variance of inter-individual variability
σ: Standard deviation of residual error
